# ECOD: An Evolutionary Classification of Protein Domains

**DOI:** 10.1371/journal.pcbi.1003926

**Published:** 2014-12-04

**Authors:** Hua Cheng, R. Dustin Schaeffer, Yuxing Liao, Lisa N. Kinch, Jimin Pei, Shuoyong Shi, Bong-Hyun Kim, Nick V. Grishin

**Affiliations:** 1 Howard Hughes Medical Institute, University of Texas Southwestern Medical Center, Dallas, Texas, United States of America; 2 Departments of Biophysics and Biochemistry, University of Texas Southwestern Medical Center, Dallas, Texas, United States of America; Stockholm University, Sweden

## Abstract

Understanding the evolution of a protein, including both close and distant relationships, often reveals insight into its structure and function. Fast and easy access to such up-to-date information facilitates research. We have developed a hierarchical evolutionary classification of all proteins with experimentally determined spatial structures, and presented it as an interactive and updatable online database. ECOD (Evolutionary Classification of protein Domains) is distinct from other structural classifications in that it groups domains primarily by evolutionary relationships (homology), rather than topology (or “fold”). This distinction highlights cases of homology between domains of differing topology to aid in understanding of protein structure evolution. ECOD uniquely emphasizes distantly related homologs that are difficult to detect, and thus catalogs the largest number of evolutionary links among structural domain classifications. Placing distant homologs together underscores the ancestral similarities of these proteins and draws attention to the most important regions of sequence and structure, as well as conserved functional sites. ECOD also recognizes closer sequence-based relationships between protein domains. Currently, approximately 100,000 protein structures are classified in ECOD into 9,000 sequence families clustered into close to 2,000 evolutionary groups. The classification is assisted by an automated pipeline that quickly and consistently classifies weekly releases of PDB structures and allows for continual updates. This synchronization with PDB uniquely distinguishes ECOD among all protein classifications. Finally, we present several case studies of homologous proteins not recorded in other classifications, illustrating the potential of how ECOD can be used to further biological and evolutionary studies.

## Author Summary

Protein structural domain databases offer a vital resource for structural bioinformatics. These databases provide functional inference for homologous structures, supply templates for structural prediction experiments, and differentiate between homologs and analogs. The rate of structure determination and deposition has increased dramatically over recent years, overwhelming the ability of current classifications to incorporate all new structures. We have developed a fast and reliable methodology for updating domain databases automatically, and created a revised hierarchy for domain classification that emphasizes evolutionary relationships. By classifying all known structures in our database with continuing automatic updates, we provide an up-to-date alternative to current resources. We illustrate several concepts that guided our classification scheme with examples of homology between domains in ECOD that are not observed in other resources.

## Introduction

The billions of proteins in extant species constitute a bewilderingly diverse protein world. To understand this world, systematic classifications are needed to reduce its complexity and to bring order to its relationships. As proteins are the products of evolution, their phylogeny provides a natural foundation for a meaningful hierarchical classification. As in the classification of species, a phylogenetic classification of proteins identifies evolutionary relationships between proteins and groups homologs (proteins that are descendants of a common ancestor) together. Because homologs generally share similar three-dimensional (3D) structures and functional properties, such a classification provides a valuable platform for studying the laws of protein evolution by comparative analysis as well as for predicting structure and function by homology-based inference.

Many protein classifications are currently available. Comprehensive sequence-based classifications such as Pfam [1] and CDD [2] are among the most popular protein annotation tools. When sequence-only methods fail to reveal more distant evolutionary links, 3D structures allow us to see further back in time, as protein structure is generally better preserved than sequence in evolution [3]. Currently, the two leading structure classifications are SCOP (Structural Classification of Proteins) [4] and CATH (Class, Architecture, Topology, Homology) [5], both of which are widely used in analyzing protein sequence, structure, function, and evolution and in developing various bioinformatics tools. CATH (http://www.cathdb.info) is largely automatic with added manual curation and emphasizes more on geometry, while SCOP is mainly manual and focuses on function and evolution.

In the SCOP [4] (http://scop.mrc-lmb.cam.ac.uk/scop/index.html) hierarchical classification, closely related domains are grouped into families; families with structural and/or functional similarities supporting common ancestry are grouped into superfamilies; superfamilies with similar 3D architectures and topologies are grouped into folds; and folds with similar secondary structure compositions are grouped into classes. Cataloging remote homologies identified by a combination of visual inspection, sequence and structure similarity search, and expert knowledge, the SCOP superfamily is the broadest level indicating homology and offers invaluable insights in protein evolution. However, SCOP tends to be conservative in assessing evolutionary relationships, and many homologous links reported in literature are not currently reflected [6], [7], [8], [9], [10], [11]. Also, the recent dramatic increase of available structures in the PDB [12] (http://www.pdb.org) hinders careful manual curation in SCOP. Recently, a new version of SCOP (SCOP2) [13] was introduced that eschews hierarchical classification in place of a network of relationships (homologous and structural), although this database has not been made current with PDB. To partially alleviate this problem, ASTRAL now offers SCOPe, a sequence-based extension of the original SCOP hierarchy [14]. Nevertheless, not a single protein classification database has kept current with the PDB database. We maintain that the most recently determined structures, especially those evolutionarily distant from classified proteins, attract the most interest and hence are the most important to classify quickly and accurately. However, automatic updates, such as those in ASTRAL, are only able to deal with easily classifiable proteins.

Here we introduce the ECOD (Evolutionary Classification Of protein Domains) database. Our goal is threefold: (1) to construct a comprehensive domain classification based on evolutionary connections, (2) to extend the realm of connections to include remote homology, and (3) to maintain concurrent updates with the PDB. Because experimental data is very sparse compared to sequence data, establishing an evolutionary-based classification scheme of structures allows for biological insight into related proteins that otherwise lack functional information. In such a scheme, close homologs admittedly represent the most relevant source of functional inference. However for most proteins, only distant homologs have been studied in detail. Fortunately, many examples have shown that analysis of proteins in the context of their distant homologs provides functional clues that advance biological research [15], [16], [17], [18]. In addition, remote homology offers deeper insights in protein evolution. In order to extend distant evolutionary relationships beyond the SCOP superfamily level in ECOD, we apply state of the art homology-inference algorithms both developed in our group [19], [20] as well as by others [21], [22], manually analyze and verify the suggested homologous links, and incorporate findings from literature. For weekly updates, we rely on a computational pipeline that automatically and confidently classifies the majority of newly released structures and flags incompletely classified and unclassifiable structures, as well as a web interface that presents those difficult to deal with structures and pre-computed data in a convenient way for rapid manual inspection and classification.

ECOD is a publicly available database (http://prodata.swmed.edu/ecod/). By focusing on remote homology and weekly updates, ECOD strives to provide a more simplified and up-to-date view of the protein world than is currently available in existing classifications. As such, ECOD is unique in combining the following features: 1) the aforementioned weekly updates, following new releases from the PDB; 2) a hierarchy that specifically incorporates sequence-based relationships in a family level of close homology; 3) a classification that reflects more distant evolutionary connections; 4) a hierarchy that lacks a SCOP-like fold level, as the definition of “fold” is often subjective [23]; 5) domain partitions for all former members of the SCOPmulti-domain protein class; and 6) combination of membrane proteins with their soluble homologs where an evolutionary relationship can be hypothesized. Theoretically, ECOD catalogs rich and up-to-date information about protein structure for the studies on protein origins and evolution; and practically, it helps homology-based structure and function prediction and protein annotation by providing a pre-compiled search database.

## Methods

We first developed a pilot version of ECOD based on SCOP 1.75 [4]. To detect remote homologies beyond the SCOP superfamily level, 40% identity domain representatives in the first 7 classes in SCOP 1.75 were retrieved from ASTRAL [24] and compared in an all-versus-all fashion. Four scores were computed for each pair: HHsearch probability [21], DALI Z-score [22], HorA combined score [20], and HorA SVM score [19]. Domain pairs with high scores were manually inspected and analyzed. The decision on whether any given pair is homologous was based on considerations of the aforementioned scores, literature, functional similarity (such as common cofactor-binding residues), shared unusual structural features [25], domain organization, oligomerization states, and disulfide bond positions. Since the SCOP superfamily level is reliable and conservative, we typically only merged SCOP superfamilies into homologous (H-) groups. In addition to merging SCOP superfamilies, we split SCOP entries with multiple domains or with duplications, and corrected rare inconsistencies in the SCOP classification. Cytoscape [26] clustering was used to aid manual analysis by displaying domains and high-scoring links. After 40% representatives were classified, other SCOP 1.75 domains were automatically mapped into the ECOD hierarchy using MUSCLE alignments [27]. Many hierarchical groups in the ECOD pilot version retained the names of their original SCOP counterparts.

**Figure 1:**
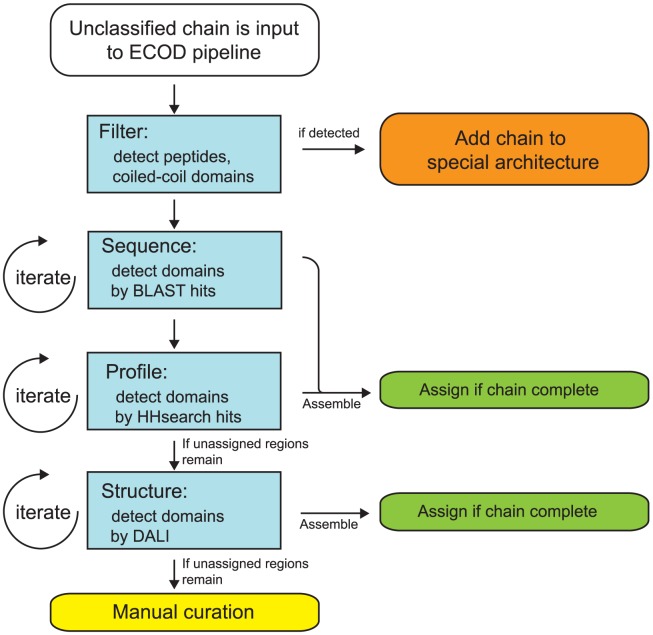
Workflow of the ECOD automatic domain classification pipeline. Unclassified structures enter from the top (white). Firstly, peptides, coiled-coils, and other unclassifiable regions are removed where possible and placed in their respective special architectures (orange). Secondly, unassigned regions of the input sequence are iteratively assigned by descending best hits from BLAST and HHsearch-based searches of ECOD databases. Assemblies of putative domains are optimized and assigned (green). If the chain is incomplete by sequence, a similar process occurs using DaliLite searches. If the query remains unclassified, it is manually curated (yellow).

Those structures not classified in SCOP 1.75 were partitioned and assigned to ECOD using a combination of sequence and structural homology detection methods. We used an iterative pipeline of three sequence homology detection methods of increasing sensitivity and decreasing specificity to partition input proteins into domains (Fig. 1). First, the input protein sequence is queried against a library of known ECOD full-length chains (containing both single-domain and multi-domain architectures) using BLAST [28],[29]. Where significant sequence similarity (E-value<2e-3) is detected to a known domain architecture with high coverage (<10 residues uncovered), the entire series of domains in the input chain was partitioned in one pass. Second, the protein sequence is queried using BLAST against a library of domain sequences. Here single-domain proteins and components of multi-domain proteins were assigned individually by sequence similarity (E-value<2e-3) and hit coverage (>80%). Finally, for detection of more distant homology, a query sequence profile was generated using HHblits [21]. This profile was used to query a database of ECOD representative domain profiles using HHsearch. Domains from the input chains could be classified by any combination of the three sequence-based methods (chain BLAST, domain BLAST, or domain HHsearch). Following partition, a boundary optimization procedure based on the structural domain parser, PDP, was run to eliminate small interstitial gaps between assigned domains and at termini [30].

Input protein chains with a set of detected domains with full residue coverage from the sequence pipeline were considered to be complete. Domains from these chains were then assigned to the ECOD hierarchy broadly using the classification of their hit domain. Following this assignment a combination of HMMER/Pfam and HHsearch-based clustering was used to finely tune family assignments [1], [31]. Domains were clustered into F-groups by Pfam where confident HMMER3-based assignments could be found. Where domains had no confident Pfam assignment, all-versus-all HHsearch-based complete linkage clustering was used to generate an F-group [32] where all domains shared 90% HHsearch probability. We specifically designate provisional representatives in F-groups where no member shares close homology with a representative ECOD domain for manual examination. Input protein chains that could not be fully assigned by the sequence pipeline were passed to the structural pipeline.

If a protein chain could not be assigned by the sequence pipeline, it was queried against a library of representative ECOD domain structures using DaliLite [33]. Domains were assigned where significant structural similarity existed to a known ECOD domain and where the aligned region passed a simple BLOSUM-based alignment score [34]. As in the sequence pipeline, the boundaries of structurally assigned domains were optimized, and those chains that could be completely assigned (100% residue coverage) were added to the classification. Where a chain could not be completely assigned, it was passed to the manual curators for boundary refinement or assignment. As we neared completion of the PDB, the need for structural search decreased as the number of remaining structures was small enough to manually curate.

Difficult structures that could not be completely and confidently classified by the pipeline required manual curation. We first inspected the mapping suggested by the pipeline. Oftentimes, the suggested mapping was correct for most or part of the query structure, and we typically accepted this mapping but modified the domain boundaries. For other queries where the suggested mapping was wrong or absent, we used HorA server [20] to search for remote homologs. In evaluating HorA results, we applied the same considerations used in developing the ECOD pilot version to determine homology between a query and a hit. When a homologous hit with similar topology could be found, the query was classified into the same T-group as the hit; when a homologous hit with different topology could be found, the query was classified in a new T-group but the same H-group as the hit; when only a possibly homologous hit with similar overall structure could be found, the query was classified in a new H-group but the same X-group as the hit; when no possible homologs can be identified, the query is classified in a new X-group by itself (see Results and Discussion for a description of the ECOD hierarchy). To facilitate manual analysis, we developed a web interface that presented relevant information in a clear format as well as recorded and incorporated feedback and annotations from manual curators.

## Results/Discussion

ECOD is a hierarchical classification of domains based on their evolutionary relationships. Focusing on remote homology, ECOD organizes domains into very broad homologous groups. At the same time, ECOD families address closer evolutionary relationships, detectable at a sequence level. Most importantly, ECOD is comprehensive and up-to-date, including all entries in the PDB and updating weekly, thus uniquely providing researchers with the most current classification of protein domains at both distant and close homology levels.

### Database Description

**Figure 2:**
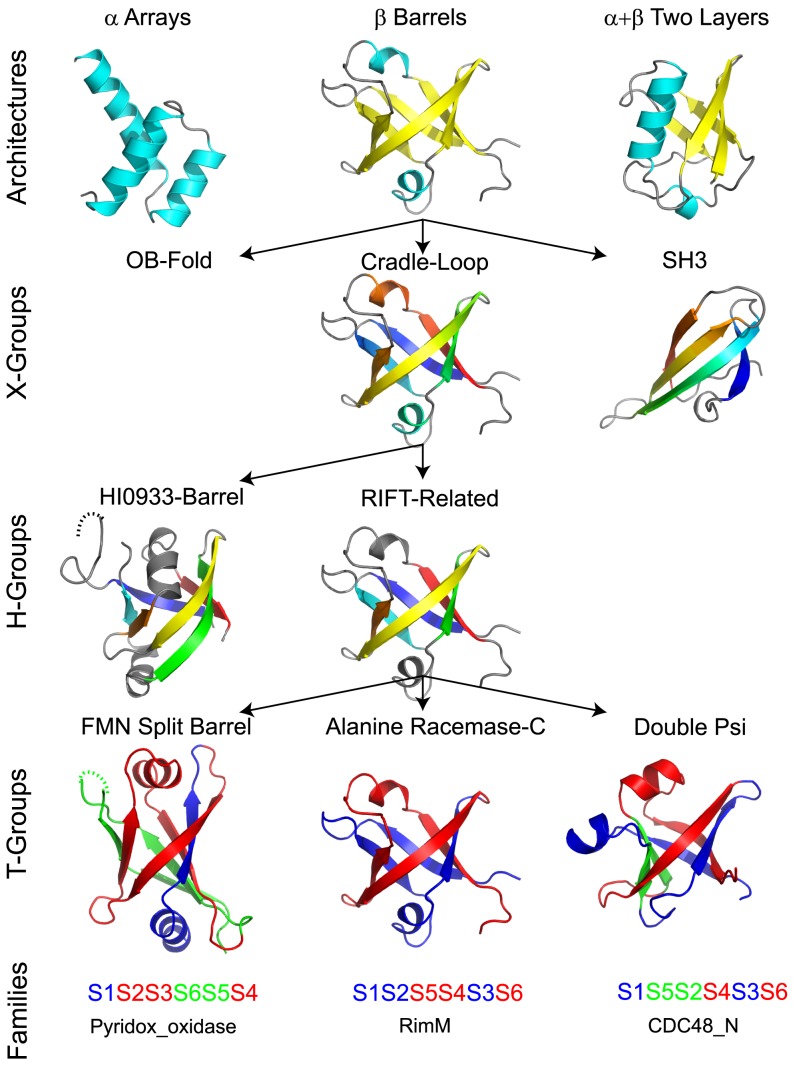
Hierarchical levels of ECOD. Domains placed within the same Architecture share similar secondary structure content (helix, cyan; sheet, yellow) and geometric arrangement. Domains placed within the same X-group share similar structure but lack a convincing argument for homology (vs. analogy), while those placed within the same H-groups are homologous. X- and H- group structures are colored in rainbow by consecutive secondary structure elements. T-groups distinguish homologous domains with notable differences in topology, such as the illustrated Rift-related metafold [18]. Rift-related half-barrels (colored blue and red) are consistent among the domains, but permutations and strand swaps (green) modify the topology.

ECOD is a hierarchical classification with five main levels (Fig. 2, from top to bottom): architecture (A), possible homology (X), homology (H), topology (T), and family (F). The architecture level (A) groups domains with similar secondary structure compositions and geometric shapes. The possible homology level (X) groups domains where some evidence exists to demonstrate homology (but where further evidence is needed). The homology level (H) groups together domains with common ancestry as suggested by high sequence-structure scores, functional similarity, shared unusual features [25], and literature. The topology level (T) groups domains with similar topological connections. The family level (F) groups domains with significant sequence similarity (primarily according to Pfam, secondarily by HHsearch-based clustering).

ECOD has 20 architectures that were developed both by consulting SCOP fold descriptions and inspecting numerous structures. We note that clear-cut boundaries between architectures do not always exist and that domain assignment to an architecture is sometimes subjective. This level is introduced largely for convenience of users and does not directly correspond to evolutionary grouping. A-level lies in between SCOP class and fold and groups proteins by simple visual features such as bundles, barrels, meanders, and sandwiches. Coiled-coils, peptides, fragments, largely disordered structures, and low resolution structures were put in special architectures with no X-, H-, T-, or F-levels, as confident evolutionary classification of these structures is challenging at the moment. Nucleic acids, in addition to proteins, are kept within a special architecture and are not currently classified. Within architectures, X-groups are ordered by structural similarity between them.

The ECOD X-level groups domains that may be homologous as is frequently suggested by similarity of their spatial structures. A domain's overall structure is traditionally referred to as its ‘fold’. Fold similarity usually refers to general resemblance in both architecture and topology and can result from either common ancestry (homology) or physical/chemical restrictions (analogy) [35],[36],[37]. Both SCOP and CATH have a fold level in the hierarchy: “SCOP fold” and “CATH topology”. However, the definition of fold can be subjective [23], and fold is a geometrical concept without explicit evolutionary meaning. Therefore, ECOD generally avoids the fold concept. However, domains that share strong overall architectural and topological similarity and are possibly homologous, but which lack further evidence to exclude analogy, are attributed to the same X-group but different H-groups. The conceptual difference between ECOD X-group and SCOP fold can be shown, for example, in the classification of domains with a ferredoxin-like topology. In SCOP, the ‘Ferredoxin-like’ fold is a large assembly of various superfamilies that share the (βαβ)×2 topology. Among all these superfamilies, 4Fe-4S ferredoxins seem unique for their small size and cysteine-rich nature (cysteines are used to coordinate the Fe-S clusters). Thus we suspect 4Fe-4S ferredoxins have an independent evolutionary origin and keep 4Fe-4S ferredoxins and other superfamilies in separate X-groups. On the other hand, although domains in the SCOP fold ‘Ribosomal proteins S24e, L23 and L15e’ do not have the ferredoxin-like (βαβ)×2 topology, their structures can easily be transformed into that topology by a circular permutation. Their structural similarity and functional similarity with the ‘RNA-binding domain, RBD’ superfamily in SCOP ‘Ferredoxin-like’ fold may imply homology. Therefore, ECOD classifies ‘Ribosomal proteins S24e, L23 and L15e’ and ‘RNA-binding domain, RBD’ as two H-groups in the same X-group as possible homologs. When further evidence coming either from additional sequences or 3D structures accumulates, classification decisions are adjusted to agree best with all available data.

We examined the distribution of domains mapped to SCOP folds and CATH topologies among ECOD X-groups. Of 1,799 ECOD X-groups, 598 include domains from only one SCOP fold and 564 include domains from only one CATH topology, reflecting agreement between classifications for these groups. 89 ECOD X-groups contain domains from multiple SCOP folds and 315 X-groups include domains from multiple CATH topologies. For example, the SCOP folds c.1-TIM beta/alpha-barrel and c.6-7-stranded beta/alpha barrel both contain domains mapped to the ECOD TIM beta/alpha barrel X-group. ECOD unifies such groups due to their shared structural similarity (7- versus 8- stranded) and similar locations of functional sites, but with insufficient evidence of homology to belong to the same H-group. 935 ECOD X-groups are not mapped to any SCOP fold, whereas 1,014 ECOD X-groups are not mapped to any CATH topology. The majority of these unmapped X-groups are simply due to proteins that are not classified by SCOP or CATH (722 and 872 X-groups, respectively); the remainder are shared proteins that are partitioned differently. Taken together, these results suggest that ECOD tends to merge both SCOP folds and CATH topologies into X-groups.

**Figure 3:**
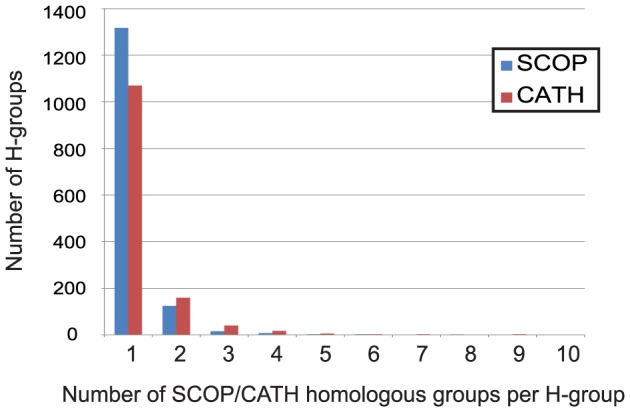
Number of ECOD H-groups containing 1 or more SCOP superfamily (blue) or CATH homologous superfamily(red). The majority contain only a single SCOP superfamily(88%) or CATH homologous superfamily (81%). The most merged (not shown) ECOD H-group is the Immunoglobulin-related domains, which contains 47 SCOP superfamilies and 81 CATH homologous superfamiles.

An ECOD H-group can contain more distant homologous links than the equivalent SCOP superfamily or CATH homologous superfamily. Although the majority of ECOD H-groups contain only a single SCOP superfamily (88%) or CATH homologous superfamily (81%), some H-groups contain many more (Fig. 3). For example, the Immunoglobulin-related and the Rossmann-related H-groups contain the most SCOP superfamiles (47 and 28, respectively) and CATH homologous superfamilies (81 and 40, respectively). Superfamilies were merged based on multiple high-scoring homologous links between domains. These merges reflect the homology between domain members of these previously split groups.

In total, 53 ECOD H-groups contain domains from two or more SCOP folds, and these H-groups contain domains from 151 unique SCOP folds, indicating that fold change in evolution of protein structures is not a very uncommon phenomenon. Similarly, 169 ECOD H-groups contain domains from two or more CATH topologies, and these H-groups contain domains from 357 unique CATH topologies. Additionally, 36 H-groups contain domains mapped to more than one CATH class, indicating homologous domains that nonetheless contain fairly different topologies.

To readily incorporate the observation that homologs can adopt different folds, ECOD has a topology (T-) level below the homology (H-) level. As a result, homologs with different topologies that SCOP necessarily separates into different folds (and thus different superfamilies) are unified in the same H-group but different T-groups in ECOD. For example, β-propellers are comprised of differing numbers of repeated β-meanders, all of which are evolutionarily related. The five different beta-propeller folds outlined in SCOP are organized in ECOD into a single H-group, with child T-groups for domains with differing number of blades [38]. Also, the domain contents of 11 SCOP folds are organized into multiple T-groups under the Rift-related H-group in the cradle-loop barrel X-group [39]. If we find sufficient evidence for homology between these proteins this consideration results in merging not only SCOP superfamilies, but also SCOP folds.

Within T-groups, ECOD organizes domains into families based on sequence similarity. We employ Pfam as the standard for family definition. ECOD domains were attributed to Pfam families by HMMER3 [31]. Therefore, the majority of ECOD F-groups are simply Pfam families. However, not all protein domains with known structure can be attributed to the current version of Pfam by sequence similarity. Those domains are grouped into families by HHsearch as outlined in Materials and Methods. As a result, ECOD contains 8,947 F-groups, 7,156 of which can be mapped to Pfam families, and 1,622 composed of homologous domains not mapped to any Pfam family.

### Summary Statistics of ECOD

Summary statistics for the ECOD database as of July 31st^th^, 2013 (version 22b) are presented in Table 1. The majority of the 317,021 domains in ECOD were assigned automatically to a smaller set of 15,969 manually curated domain representatives. Domains in ECOD were derived from five sources: 1) domains originally in SCOP ASTRAL40, inherited and reclassified manually in ECOD (11,462), 2) domains originally in SCOP, but not in the ASTRAL40 set, mapped by MUSCLE alignment with their ASTRAL representative (98,702), 3) novel domains not contained in SCOP, usually from chains deposited to the PDB in the intervening period between the release of SCOP v1.75 and ECOD, manually curated and added to the representative set (4,373), 4) domains automatically added to ECOD by detection of homology by pairwise sequence or structure search (153,381), and 5) domains added to ECOD by MUSCLE alignment of non-representative sequences to closely related ECOD representatives (48,817). The vast majority of domains classified in ECOD have been added by automatic methods. ECOD provides for domains which are assembled from multiple PDB chains, either due to photolytic cleavage (i.e. order-dependent assembly) or obligate multimers (i.e. order-independent assemblies). For order-independent assemblies, we distinguish between those domains where the assembly is primarily relevant for display, or appears to be biologically necessary. These are fairly rare in the database; only 132 representative order-independent assemblies have been defined. At the time of writing, 100% of PDB depositions could be accounted for in the ECOD classification (including those members of the special architectures).

**Table 1:** Summary statistics of ECOD v22b (July 31 2013).

Level	Population
**Architectures**	20
**X-groups**	1,799
**H-groups**	2,279
**T-groups**	2,865
**F-groups**	9,013
**Manual representatives**	15,969
**Domains**	317,021
**95% nonredundant domains** 1	50,305
**PDB structures**	93,663
**Peptide chains**	239,303

1 domains were filtered using BLASTCLUST with a 95% sequence identity threshold and 90% length cutoff.

**Figure 4:**
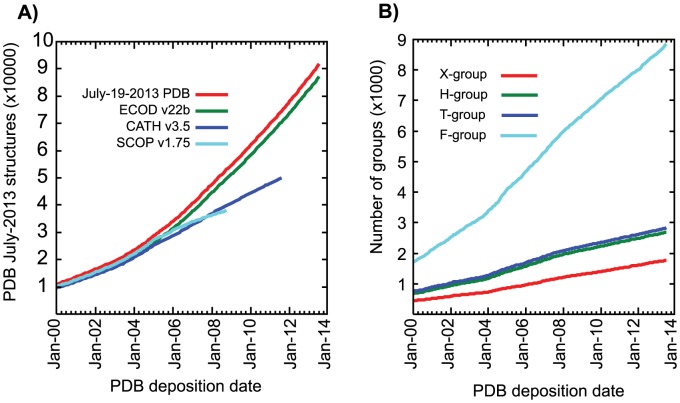
Classification of ECOD and ECOD hierarchical levels with respect to the PDB and other classifications. A) A cumulative sum of PDB release dates from Jan-2000 to Jan-2014 (red) compared to classified PDB depositions in ECOD (green), SCOP (cyan), and CATH (blue). Any deposition with at least one domain classified is counted. ECOD consistently classifies more structures than SCOP and CATH and is more up-to-date. b) The cumulative sum of PDB deposition dates in ECOD hierarchical levels. Each group is classified once by its oldest deposition. The number of new levels increases consistently over time over the 2000 to 2014 time period.

We also compare ECOD to the most recent releases of SCOP and CATH. ECOD, SCOP, and CATH differ in domain partition strategy, classification hierarchy, and simply in the number of structures considered. At the time of writing, ECOD classifies 93,663 PDB depositions containing 239,303 protein chains, SCOP 1.75 contains 38,221 PDBs and 85,141 chains, and CATH v3.5 contains 51,334 PDBs and 118,792 chains. Of those chains classified in ECOD that are not in SCOP (and not in a special architecture), 137,794 were automatically classified and 2,484 were classified manually. Of those chains classified in ECOD, but not in CATH (and not in a special architecture), 106,474 were automatically classified and 2,521 were classified manually. The growth of the PDB over time is compared to the number of structures classified in ECOD, CATH, and SCOP (Fig. 4(a)). The difference between the number of structures in the PDB and those in the main architectures of ECOD can be primarily accounted for by the number of structures contained in ECOD special architectures (i.e. coiled-coil, peptide, non-peptide polymers, and low-resolution structures that could not be classified by sequence). The growth of the hierarchical levels from 2000–2013 indicates that although evolutionary distinct groups (i.e. X- and H- groups) are being discovered at a steady pace, the predominant source of new domains in ECOD is from sequence families (F-groups) being associated with existing homologous groups (Fig. 4(b)).

### Classification of Weekly PDB Structure Updates

**Figure 5:**
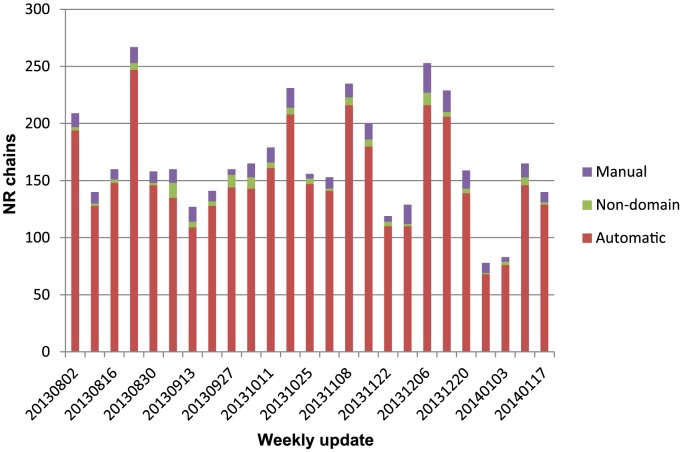
Classification methods used for non-redundant (NR) chains for weekly ECOD updates. “Automatic” chains could be completely and confidently classified by domain pipeline and required no manual intervention. “Manual” chains were at best partly classified by software and required manual curation (i.e. some domain boundaries could not be properly detected or some domains could not be reliably classified using sequence methods). Non-domain” chains contained peptides, coiled-coils, or other cases requiring manual curation.

Since the July 2013 version, whose statistics are presented here, the subsequent 25 weekly releases by the PDB have been automatically classified (Fig. 5). Each week, protein chains are clustered at 95% redundancy, representatives for those non-redundant chains are classified; those remaining chains are classified when the initial automatic and manual classification pass are completed. For each weekly update, the majority (∼89%) of non-redundant (<95%) chains can be partitioned and assigned automatically (134.1±40.4). Those chains that cannot be resolved automatically are manually curated. On average, 11.7±4.9 chains per week were classified as manual representatives in ECOD, whereas 5.1±3.2 were chains not containing domains (i.e. peptides, coiled-coils, or fragments) that were resolved by assignment to special categories or other methods that did not modify the hierarchy. Overall, the majority of protein chains in weekly PDB releases can be classified automatically into ECOD.

### Most Populated Homologous Groups in ECOD

**Figure 6:**
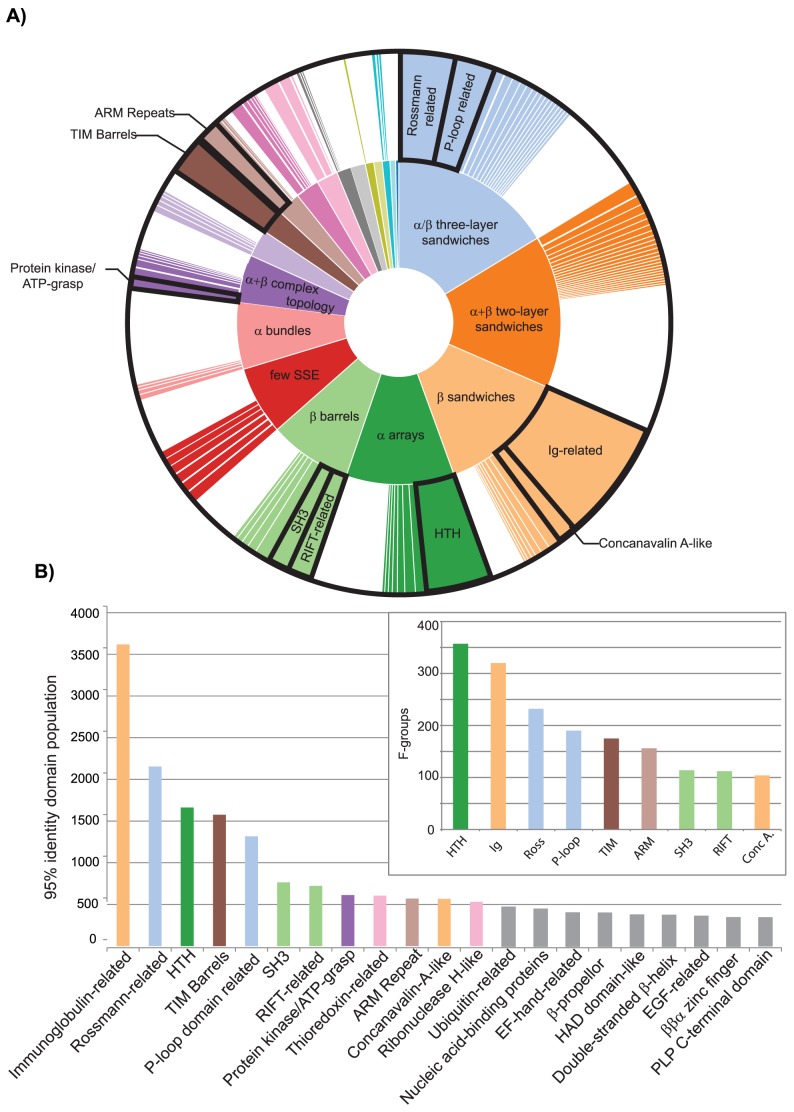
Distribution of H-groups in ECOD by architecture (a) and 95% representative domain population (b). A) H-groups are colored by architecture and sized according to their representative domain population. H-groups smaller than 0.01 radians are not displayed. Those H-groups shown in bottom distributions are labeled. B) The most populated H-groups (>500 95% representative domains) are colored by architecture. The immunoglobulin-related, Rossmann-related, and helix-turn-helix (HTH) H-groups are the most populated H-groups in ECOD. The inset shows the most populated H-groups by number of F-groups.

We analyzed the distribution of domains in hierarchical levels in ECOD. The most populated homologous groups (H-groups) are placed in context with their architecture in ECOD (Fig. 6(a)) and are also ranked by population (Fig. 6(b)). The Ig-related and Rossmann-related H-groups, in addition to containing the most merged SCOP and CATH homologous groups, are the most populated H-groups in ECOD. The merging of many previously distinct helix-turn-helix (HTH) SCOP superfamilies in ECOD boosts the population of this H-group considerably compared to its original SCOP population. The inset (Fig. 6(b)) shows those most populated H-groups by number of F-groups. Where many sequence families have been merged by distant homology, such as the RIFT-related or Immunoglobulin-related domains, H-groups will contain many F-groups. In ECOD, as opposed to SCOP or CATH, there exist fewer distinct homologous groups with related topologies, as many of these groups have been linked by homology. For example, in ECOD, there is a single Rossmann-related H-group among the most populated (top 15) groups, whereas in the most populated SCOP superfamilies or CATH homologous superfamilies, there are two (NAD(P)-binding Rossmann fold domains and SAM methyltransferases) and four (3.40.50.720, 3.40.50.1820, 3.40.50.150, and 3.40.50.2300), respectively.

**Figure 7:**
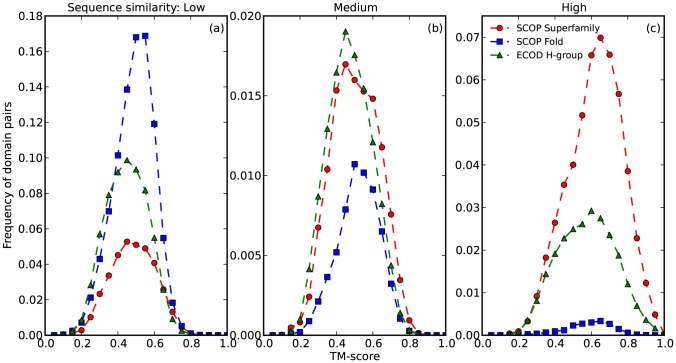
Structure similarity distribution of domain pairs from SCOP superfamily, SCOP fold and ECOD H-group, measured by TM-score. Data were grouped into three panels by sequence similarity in terms of HHsearch probability (Low: probability ≤20%, Medium: 20%<probability<90%, High: probability ≥90%) and then binned into 20 bins to calculate frequency.

We compared our H-groups to SCOP superfamilies and folds by considering sequence and structure similarity of domain pairs within each level. ECOD manual representatives and ASTRAL40 domains were evaluated by HHsearch to reflect sequence similarity and TMalign to reflect structure similarity [21], [40]. SCOP superfamilies tend to contain more close homologs that can be detected by sequence homology search methods than ECOD H-groups (Fig. 7(c)). Domains classified in SCOP folds (excluding pairs from the same superfamily) emphasize structural similarity, as the distribution is mostly populated in the low sequence similarity region and the peak shifts right compared with others (Fig. 7(a,b)). On the other hand, as ECOD H-group readily incorporates homologous links from SCOP superfamilies and also many remotely homologous relationships that were previously overlooked, its peak sizes lie between SCOP fold and superfamily in high and low sequence similarity regions. Also it is worth noting that the peak of ECOD H-group does not have the right shoulder in the intermediate sequence similarity group but has a relatively evident left shoulder in the high sequence similarity group (Fig. 7(b,c)), which potentially supports the idea that ECOD classification is homology-centric.

### Domain Partition in ECOD, SCOP, and CATH

**Figure 8:**
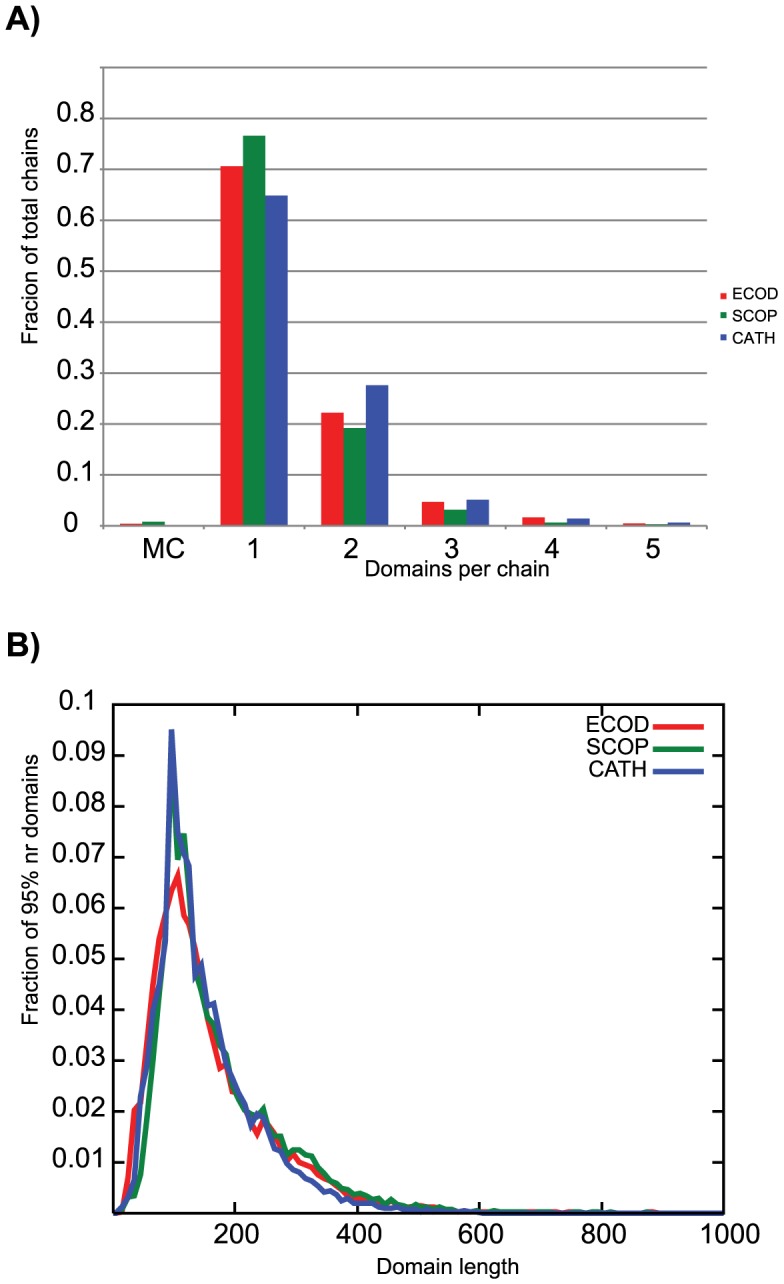
A) Distribution of domains per chain for ECOD(red), SCOP(green), and CATH(blue). Both ECOD and SCOP allow for multi-chain domains (MC), but these are a small fraction of the classification. ECOD contains slightly more single-chain domains than CATH, but less than SCOP. B) ECOD slightly favors smaller domains over SCOP and longer domains over CATH.

We compared the domain partition observed in ECOD, SCOP, and CATH. Domain partition strategy can differ markedly between classifications, depending generally on whether the presence of compact structural units or overall sequence similarity is emphasized. The number of domains per chain observed in the domain classifications is presented in Figure 8(a). ECOD splits more protein chains (29%) into multiple domains than SCOP (23%), but splits slightly less than CATH (35%). The size distribution of domains in ECOD, SCOP, and CATH was compared (Fig. 8(b)). ECOD favors slightly shorter domains than SCOP, and favors slightly longer domains over CATH, but the size distributions are very similar. These results are consistent with the differences in domain definition strategy employed by different classifications. CATH emphasizes on structural integrity of the domain and its structural separation from other domains, SCOP focuses on the occurrence of an individual domain in different domain combinations, and ECOD attempts to find a compromise between these two strategies.

### Similarity of Classification between Equivalent Domains

**Figure 9:**
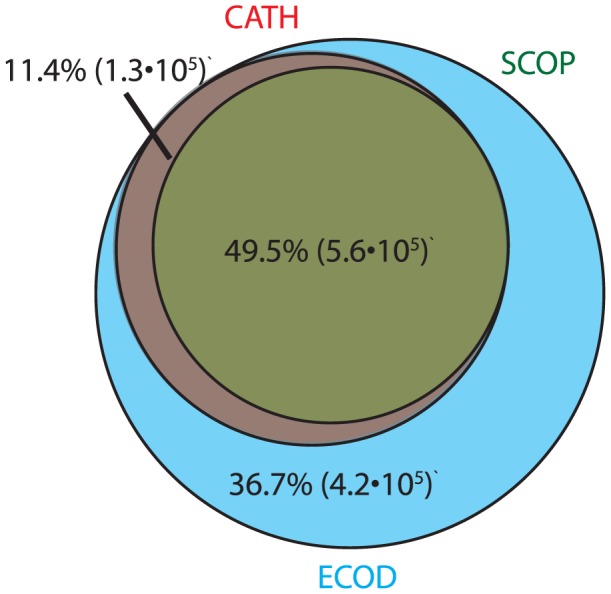
Venn diagram of the shared homologous domain pairs among those ECOD (cyan), SCOP (green), and CATH (red) nonredundant domains with similar (80%) domain ranges. A plurality of domain pairs are shared among all three classifications. A large fraction of domain pairs can solely be observed in ECOD. 11.4% of domain pairs are only shared between ECOD and CATH.

The difference in homologous links among equivalent domains was analyzed in ECOD, SCOP, and CATH. We define equivalent domains as those that share 80% residue coverage in all classifications. This subset of domains contains those domains whose partition is similar among classifications, but whose classification and homologous cluster size differ. We then analyze whether those domains that share a homologous link within one classification also share that link in other classifications. For the purposes of this analysis, only SCOP domains from canonical SCOP classes [a–d] are considered. Of the total domains in ECOD, 67,559 are defined equivalently (by 80% residue coverage) in SCOP and CATH. As many of these domains are identical or near identical in sequence, only domains with less than 95% sequence identity are used. There are 9,523 equivalent, non-redundant domains, shared among SCOP, CATH, and ECOD. Any pair of those equivalent domains belonging to the same H-group is considered to be homologous, 1,030,085 of these homologous domain pairs were observed in ECOD. Similar analysis was performed on SCOP superfamilies and CATH homologous superfamilies, where 711,894 and 680,726 homologous domain pairs were observed respectively. On average, 49.5% of domain pairs were shared between classifications, 36.6% of domain pairs were only observed in ECOD, 11.4% of domain pairs were observed only between ECOD and CATH (Fig. 9). Negligible numbers of domain pairs were observed in SCOP only, CATH only, or SCOP/CATH only. These results reflect a set in which most known homologous relationships among similarly partitioned domains are similar in ECOD as in SCOP and CATH. Additionally, ECOD catalogs many homologous relationships (among these similarly partitioned domains) that are not observed elsewhere.

SCOP recently diverged into two separate projects: SCOPe [14], which continues to update the original SCOP hierarchy using conservative automated methods, and SCOP2 [13], which is a dramatic reimagining of protein classification away from a hierarchal tree to a network model. We compared both of these more recent SCOP databases to ECOD. SCOP2 (February prototype version) eschews the traditional classification model; individual residues can be classified at multiple nodes in the network. We considered all SCOP2 domains, regardless of level, in comparison to ECOD. Of 995 PDBs and 1010 chains classified in SCOP2, equivalent domains were found in 725 PDBs and 732 chains. 70% of SCOP2 domains defined at the sequence family level were ECOD-equivalent. Conversely, only 56% of SCOP domains defined at the structural fold level (340/605) and sequence superfamily (272/482) level were equivalent to an ECOD domain. Only 61 of 257 domains defined at the hyperfamily (HF) level, are equivalent any domain in ECOD. Only 121 of 2,973 ECOD H-groups in this comparison were mapped to any domain in SCOP2. In general, the incomplete coverage of SCOP2 makes general statements about differences from ECOD premature.

SCOPe (v2.03-stable) uses a conservative automated method to add domains to the SCOP v1.75 hierarchy. Since both ECOD and SCOPe were derived from SCOP v1.75, we were particularly interested in classification of recent chains. ECOD v49 and SCOPe v2.03 (stable) contain 261,704 and 163,351 domains from shared protein chains, respectively. Of those SCOP-mapped ECOD domains, 94,292 were derived from SCOP v1.75 domains and 57,929 were independently classified. 27,142 ECOD domains derived from SCOPe shared chains do not map to any SCOPe domain, reflecting direct differences in domain partition strategy between SCOPe and ECOD. 1,493 SCOPe domains arise from structures classified only by SCOPe, but these structures are dominated by peptides and coiled-coils, regions that are not classified as domains by ECOD. 9,164 ECOD domains were derived from SCOP v1.75 domains, but are not mapped to SCOPe. These domains were generally the result of subdivision of a larger SCOP domain. There is a core set of domains that are shared by SCOPe and ECOD, both arising due to their shared origin and also due to independent classification of more recent domains. The differences in domain partition likely arise from differences in treatment of domain duplication and subdomains and are a potential target for further study.

### Unique Hierarchical Levels in ECOD

**Figure 10:**
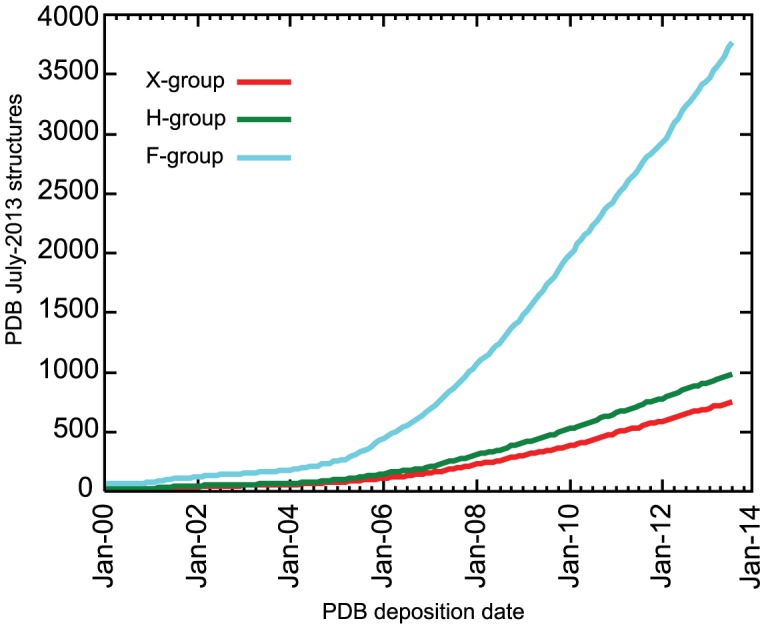
Growth of ECOD groups with no mapping to SCOP or CATH over time. Growth of all groups increases as proportion of PDBs classified by SCOP or CATH decreases. Unmapped H-groups represent a significant fraction of total ECOD H-groups. Unmapped X-groups are potentially interesting cases of novelty.

**Figure 11:**
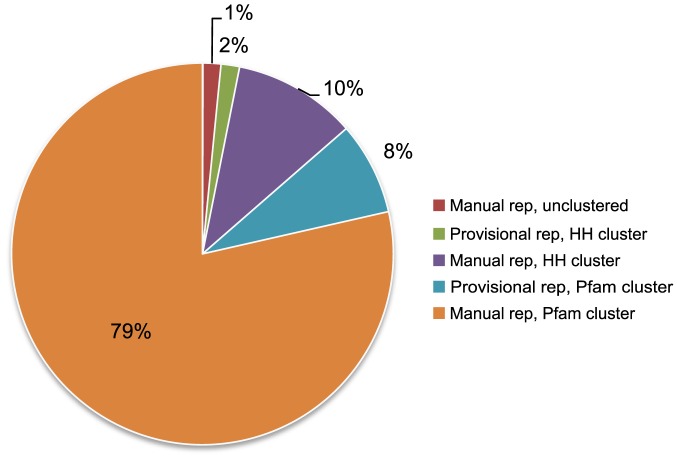
Representative domains in ECOD classified by type (manual or provisional) and cluster type (Pfam, HH, or unclustered). Manual representatives have been inspected by a curator and assignment to the hierarchy has been verified. Provisional representatives contain no close homologous link to a manual representative and cluster separately into a Pfam- or HH-based cluster. Unclustered representatives are either awaiting clustering or cannot be clustered due to some technical problem. The majority of representatives in ECOD are manual Pfam representatives (79%), followed by manual HH-clustered representatives (10%) and provisional Pfam representatives (8%).

We consider the growth-over-time analysis of ECOD in the context of the domain mapping between ECOD, SCOP and CATH (Fig. 10). Where an ECOD level (X-, H-, T-, or F-group) contains one or more domains with a mapping to a SCOP or CATH domain, we remove that level from consideration. We then re-plot the growth over time of ECOD using only those groups that contain no mapping to domains from other classifications. There is marked increase in novel ECOD classifications beginning in January 2005. The most recent deposition dates contained in SCOP 1.75 and CATH 3.5 are October 2008 and August 2011, respectively. However, the increase in novel classifications begins when the total PDBs and the PDBs classified in SCOP and CATH begin to diverge. The novel H-groups in ECOD (997) account for nearly 45% of total H-groups in ECOD. Those F-groups with no manual representative (where all domains were assigned automatically) are assigned a provisional manual representative. The majority of these automatically generated F-groups with no manual representative are derived from known Pfam families (Fig. 11). The increase in novel hierarchical levels in ECOD clearly demonstrates the value of an updated and comprehensive domain classification.

### Examples of Homologous Links in ECOD

In addition to comparison of broad statistics of ECOD, we also present three examples of homologous relationships recorded in ECOD but not observed in other classifications. We consider any two homologous domains to have a “homologous link.” Firstly, we demonstrate the homologous link between SAM-dependent methyltransferases and NAD(P)-binding Rossmann-fold domains. These domains share topological connections, but a strand invasion causes them to bear distinct topologies, nonetheless, they share strong sequence similarity. Secondly, we show how members of the cysteine-rich domains of Frizzled share homology with other domain families that can primarily be detected by conserved patterns of cysteines. Finally, we describe a novel homologous link between Duf371 and the GutA-like PTSIIA component domain families within the topologically diverse cradle-loop barrel X-group. Each of these distinct examples demonstrates how the particular focus of distant homology in ECOD can reveal previously unknown relationships.

### SAM-Dependent Methyltransferases and Rossmann-Fold Domains

**Figure 12:**
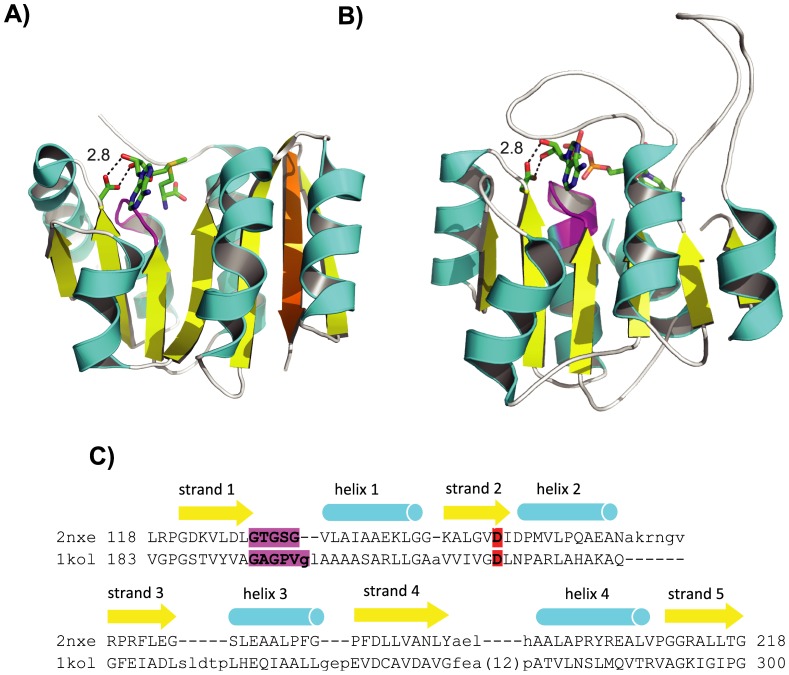
SAM MTases and Rossmann domains. (A) SAM MTases as represented by ribosomal protein L11 methyltransferase complexed with SAM (PDB 2nxe). (B) Rossmann domains as represented by formaldehyde dehydrogenase complexed with NAD (PDB 1kol). In (A) and (B), helices are colored in cyan, strands in yellow, and loops in white. The additional strand 7 in SAM-MTase is colored in orange. The respective cofactor, SAM or NAD, is shown in sticks. The Gly-rich loop beneath the cofactor is colored in magenta. The conserved Asp or Glu that forms hydrogen bonds with the adenosine ribose hydroxyls is shown in sticks. Diagrams are made by Pymol (The PyMOL Molecular Graphics System, Schrödinger, LLC. http://www.pymol.org/). (C) Manually modified DALI [22] alignment between the two domains shown in (A) and (B). Starting and ending residue numbers are labeled before and after the alignment. β-strands and α-helices are labeled numerically and shown in arrows and cylinders respectively above the sequence alignment. The Gly-rich loop is highlighted in magenta, and the conserved Asp or Glu is highlighted in red.

ECOD contains many homologous links that are not recorded in other classification databases. One example is the relationship between S-adenosyl-L-methionine-dependent methyltransferases (SAM MTases) and NAD(P)-binding Rossmann-fold domains (Rossmann domains). SAM MTases methylate a wide range of substrates using the methyl group donated by the cofactor SAM, which is comprised of an adenosine nucleoside and a methionine amino acid joined together. Rossmann domains are found in many oxidoreductases that transfer electrons between substrates and the cofactor NAD(P), which is comprised of a nicotinamide nucleotide and an adenine nucleotide joined together. Thus, SAM and NAD(P) share the adenosine part but differ in the other half, and the two enzyme superfamilies exploit the dissimilar parts of the cofactors to catalyze different reactions [41], [42], [43]. SAM MTases have a consensus structure of a 7-stranded β-sheet sandwiched between connecting α-helices (strand order 3214576 with strand 7 antiparallel to the other six strands, Fig. 12(a)) [44]. Rossmann domains have a consensus structure of a parallel 6-stranded β-sheet sandwiched between connecting α-helices (strand order 321456, Fig. 12 (b)) [45]. Thus, the SAM MTase structure can be viewed as Rossmann domain structure with a strand invasion: the additional strand 7 is inserted into the β-sheet between strands 5 and 6.

**Figure 13:**
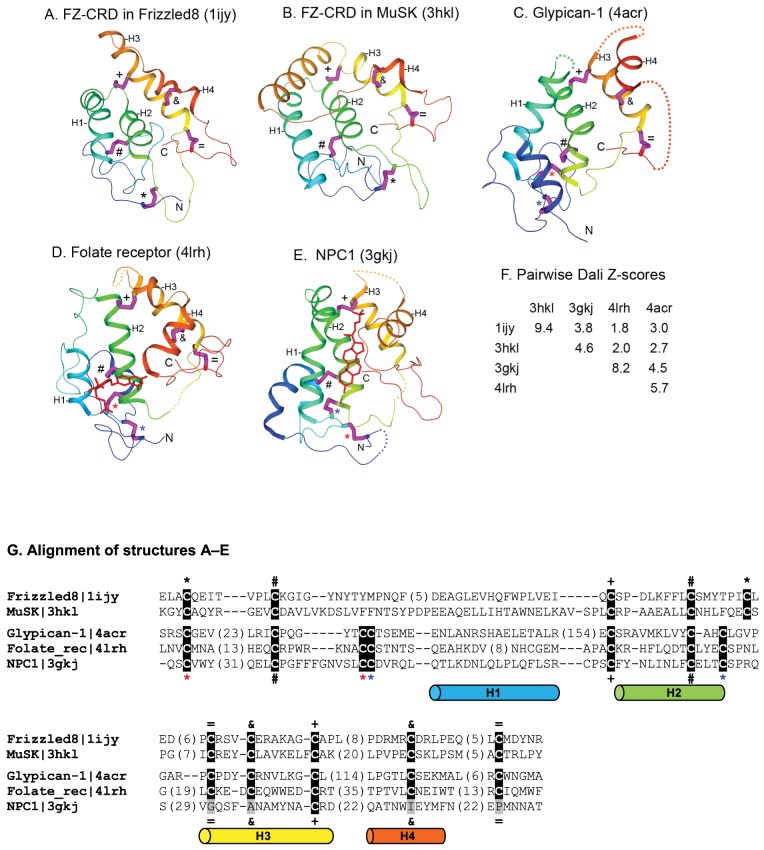
Structures of homologous members of the FZ-CRD (A,B), glypican (C), folate receptor (D), and NPC1 (E). Conserved disulfide bonds are shown in pink sticks with labels by their sides. Four core helices are labeled H1–H4. N- and C-termini are shown. Homology detected by distinct cysteine residue patterns was used as the basis for merging these families into a homologous group (H-group) in ECOD. F. Pairwise Dali Z- scores between pairs of the structures. G. Multiple sequence alignment of the structures shown, with conserved cysteines highlighted on black background. Cysteines forming a disulfide bond are labeled by the same sign for FZ-CRDs from Frizzled8 and MuSK (line above the sequences) and glypican, folate receptor and NPC1 (line below the sequences). Four core helices (H1–H4) are shown below the alignment in cylinder representation.

In SCOP, SAM MTases and Rossmann domains are classified in different folds (and therefore different superfamilies, SAM MTases: c.66.1; Rossmann domains: c.2.1), while in CATH, they are in the same topology group but different homology groups (SAM MTases: 3.40.50.150; Rossmann domains: 3.40.50.720). Although both SCOP and CATH indicate by their classification that SAM MTases and Rossmann domains are not homologous, literature suggests that they are actually related [46], [47]. As noted in reference [41], the overall structural similarity between SAM MTases and Rossmann domains is reflected in the observation that they are reciprocally the closest DALI hits to each other. In addition, SAM MTases and Rossmann domains bind their respective cofactors in a very similar fashion: the common adenosine part of the cofactors resides on top of a glycine-rich loop between the first strand and the first helix, and the adenosine ribose hydroxyls usually form hydrogen bonds with a conserved aspartate or glutamate residue at the end of the second strand (Fig. 12(a,b)) [41], [45], [46]. Indeed, the sequence-based homology detection algorithm HHsearch [21] and server HHpred [48] also provide statistical evidence that SAM MTases and Rossmann domains are related. In Cytoscape [26] display of SCOP domains and high-scoring links between them, numerous links with HHsearch probability above 90% exist between SAM MTases and Rossmann domains. In HHpred runs, for instance, when the Rossmann-domain in formaldehyde dehydrogenase (SCOP domain d1kola2, classified in c.2.1, Fig. 12(b)) is submitted as query to search against scop95_v1.75B database with secondary structure scoring turned off, the top hits within the same c.2.1 superfamily are followed by a region of mixed hits from both Rossmann domains superfamily (c.2.1) and SAM MTases superfamily (c.66.1). The highest-scoring hit from SAM MTases superfamily is hypothetical protein TM0748 (SCOP domain d1o54a_) with a 97.89% probability, E-value 9.4e-09, and identities 17% out of 110 aligned residues. Another SAM-MTase, ribosomal protein L11 methyltransferase (SCOP domain d2nxca1, Fig. 13(a) shows a same domain d2nxea1 with SAM bound), is detected with a 97.33% probability, E-value 3.4e-07, and identities 23% out of 102 aligned residues. Based on overall structural similarity, cofactor-binding resemblance, the number of confident homologous links observed between domains in each group, and statistically significant sequence similarity, ECOD classifies SAM MTases and Rossmann domains in the same homology (H-) group but different topology (T-) groups.

### Cysteine-Rich Domains in Frizzled, NPC1, Folate Receptor, and Glypicans

Frizzled receptors possess an extracellular cysteine-rich domain (FZ-CRD) for binding the Wnt ligands. FZ-CRD, as a mobile evolutionary module, has been found in other proteins such as the Smoothened receptor in Hedgehog signaling, secreted Frizzled-related proteins (SFRPs), and receptor tyrosine kinases MuSK and ROR. Sequence similarity searches and structural comparisons revealed distant similarities among FZ-CRD, Niemann-Pick type C1 protein (NPC1) that functions in cholesterol transportation, folate receptors and riboflavin-binding proteins (FRBPs) [17]. Recently, the core structures of two glypicans, proteoglycan molecules that regulate the signaling of a number of morphogens, were solved [49], [50]. Interestingly, comparative structural analyses suggested that glypicans also contain a cysteine-rich domain homologous to FZ-CRD and NPC1 [51]. Domains homologous to FZ-CRD and NPC1 have a wide distribution in eukaryotes, as they were also found in a number of other protein families currently without available structures, such as Hedgehog interacting proteins (HHIPs), RECK (reversion-inducing-cysteine-rich protein with Kazal motifs) proteins, the calcium channel component Mid1 in fungi, and the uncharacterized FAM155 proteins in metazoans [51].

The ECOD database unifies available structures of FZ-CRD, NPC1, folate receptor, and glypicans in one homologous group based on compelling sequence and structural similarities among them [17], [51]. These domains share similar disulfide bond patterns and adopt a similar overall structure fold with four core α-helices. Structural studies of three FZ-CRDs, in mouse Frizzled8 (Fig. 13(a)) [52], mouse SFRP3 [52], and rat MuSK [53] (Fig. 13(b)), revealed a common fold mainly consisting of four core α-helices (H1–H4 in Fig. 13). These FZ-CRD domains exhibit conservation of ten cysteines with a general pattern of ‘C*C*CX_8_CX_6_C*CX_3_CX_6,7_C*C*C’ (C: conserved cysteine; *: a variable number of residues, X*_n_*: *n* residues, and X*_m_*_,*n*_: *m* to *n* residues) (Fig. 13(g)). The disulfide connectivity patterns among the ten conserved cysteines are C1–C5 (between the first and fifth conserved cysteines), C2–C4, C3–C8, C6–C10, and C7–C9 (marked by black *, #, +,  = , and & signs, respectively in Fig. 13(a,b and g)). The homologous cysteine-rich domain in glypicans possesses 12 conserved cysteines with a similar pattern of ‘C*C*CC*CX_8_CX_2,3_C*CX_3_CX_6_C*C*C’ (Fig. 13(c,g)) similar to that of the FZ-CRD. Such a pattern and disulfide bond connectivity (C1–C3, C2–C5, C4–C7, C5–C10, C8–C12, and C9–C11) (Fig. 13(g)) in glypicans are also seen in the structures of FRBPs including a folate receptor [54] (Fig. 13(d)) and a riboflavin-binding protein [55]. The structure of the cholesterol-binding domain of NPC1 [56] possesses eight of these 12 conserved cysteines, while lacking two disulfide bonds formed by C8–C12 and C9–C11 in glypicans, FRBPs (Fig. 13(c)). Together, the homologous cysteine-rich domains in Frizzled, NPC1, FRBP, and glypicans define a diverse superfamily of extracellular protein domains with an ancient eukaryotic origin and potential ligand-binding activities. Duplication and divergence of such a domain have resulted in a number of families with various functions in eukaryotic membrane transport and signaling. Despite overall similarity in fold and disulfide connectivity patterns, high structural divergence, reflected by low Dali Z-scores (Fig. 13(f)), was observed between some of these structures. The ECOD classification of this homologous group of proteins includes recently solved structures such as glypicans [49], [50] and the folate receptor [54]. In contrast, both the SCOP and CATH databases only have structures of FZ-CRDs from Frizzled receptors and SFRPs and do not include the structures of FZ-CRD of MuSK [53], NPC1 [56], glypicans, and folate receptor (although the sequence of MuSK is classified in the related CATH FunFam database).

### Unique Structure Features Link Duf371 and PTSIIA

**Figure 14:**
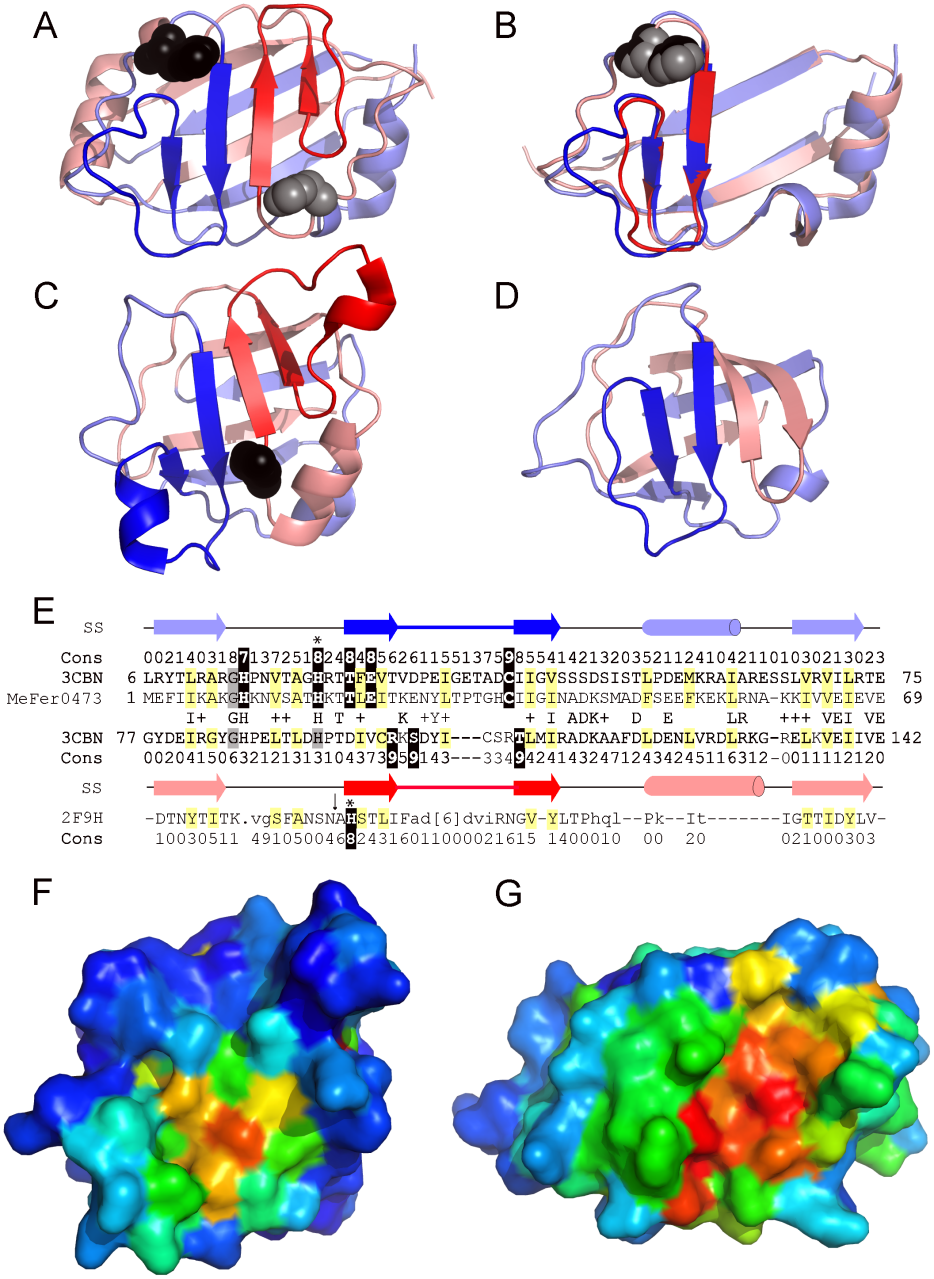
ECOD recognizes novel evolutionary relationships. A) Duf371 (3cbn) forms an 8-stranded β-barrel from intertwined β-strands of a tandem structural duplication. The N-terminal half (blue shades) includes an overside connection between adjacent β-strands (blue) that follows a conserved His (black spheres). The symmetrically related C-terminal half (red shades) includes a similar overside connection (red) following a less conserved His (gray spheres). B) The Duf371 C-terminal repeat (salmon) is rotated about the Z-axis to superimpose (RMSD 1.3) with the N-terminal repeat (slate). C) The GutA-like PTS system IIA component (2f9h) forms a similar duplicated β-barrel. An invariant His in the C-terminal half likely represent the PTS IIA phosphorylation site. D) The PK β-barrel domain-like fold (1pkla1) displays a similar intertwined topology, but retains only a single overside connection (blue) in the N-terminal half. E) PSI-BLAST alignment of the Duf371 repeats detected with Mefer0473 sequence supports the duplication event, with sequence similarities indicated between N-terminal and C-terminal halves. A structure-based alignment of the 2F9H C-terminus is included below. Structural elements (arrow for strand and cylinder for helix) and conservations (calculated by Al2Co [59]) are indicated above/below the corresponding sequences. Conserved positions are highlighted yellow (mainly hydrophobic) and black (polar). Surface representations of F) PTSIIA in the same orientation as in panel C and G) Duf371 in the rotated orientation of panel B are colored in rainbow according to conservation, from blue (less) to red (more).

ECOD establishes a previously unrecognized homologous link between a domain of unknown function (Duf371, PDB:3cbn) and the bacterial GutA-like PTS system glucitol/sorbitol-specific IIA component (PTSIIA, PDB:2f9h). While Duf371 is absent in SCOP, CATH classifies its fold (2.60.120.630) separately from that of PTSIIA (2.40.33.40). Duf371 forms an 8-stranded β-barrel from the intertwined β-strands of a tandem duplication (Fig. 14(a)). The duplicated structure elements can be superimposed (RMSD 1.3 Å), with a conserved His-containing motif from the N-terminal repeat overlapping a somewhat less conserved His-containing motif from the C-terminal repeat (Fig. 14(b)). Accordingly, PSI-BLAST [57] provides sequence evidence for this duplication, with both halves of the Duf371 query (PBD:3cbn, gi|169404770) confidently detecting the *Methanocaldococcus fervens* sequence Mefer0473 (3cbn[A:6-141] hits Mefer0473 with E-value 1e-30 in the first iteration, and 3cbn C-terminal range [A:77-142] hits with E-value 0.003 in second iteration). PTSIIA adopts a similar β-barrel topology as Duf371 and is noted in SCOP as consisting of two intertwined structural repeats (Fig. 14(c)). The overside connections between adjacent β-strands of the duplicated structure motifs in Duf371 and PTSIIA do not frequently appear in barrel architectures and distinguish the two folds.

A similar overside connection occupies the N-terminal half of pyruvate kinase (PK) β-barrel domain-like folds (ECOD/SCOP domain e1pklA1/d1pkla1). The PK barrel adopts a duplicated topology like PTSIIA and Duf371, although it lacks the C-terminal overside connection. The absence of this structural element in PK results in a 7-stranded β-barrel (Fig. 14(d)). The PK barrel half lacking the overside connection forms a ββxβ unit characteristic of the cradle-loop barrel metafold, which encompasses homologous folds of different topologies [39]. Based on the presence of a GD-box motif, the PK barrel was described as related to ancient RIFT-related folds (i.e. translation protein EF-Tu PDB: 1d2e) by a strand invasion of the N-terminal ββxβ unit that creates the overside connection [39]. Interestingly, the GD-box was also identified in both halves of PTSIIA (N-terminal GD and C-terminal GT) [58], but is not present in Duf371.

Structural similarity between Duf371 and PTSIIA is evidenced by their being reciprocal top Dali hits of each other (Dali Z-score 6), with the next best hits being to various RIFT-related homologs such as the PH barrel. The resulting structural alignment of the PTSIIA C-terminal sequence with both Duf371 sequence repeats is shown in Figure 14(e). A conserved C-terminal PTSIIA His residue (highlighted in black) marks the potential active site (Fig. 14(f)). Although the corresponding site in the Duf371 C-terminal repeat sequence is less conserved, an almost invariant threonine in the Duf371 N-terminal repeat aligns to the proposed PTSIIA functional site. Accordingly, the two folds may be related by a circular permutation of the structural repeats, maintaining a similar conserved active site position within the symmetry-related fold of Duf371 (Figure 14(g)). Considering the distinct topology of the duplicated structural motifs containing unusual overside connections, the unique way the two motifs tangle together to form an 8-stranded barrel, and the maintenance of similar active site positions, ECOD classifies PTSIIA and Duf371 as homologs in the same T-group within the RIFT-related H-group.

### Conclusions

The ECOD database summarizes our views about partitioning of protein structures into domains and this evolutionary classification is a comprehensive resource for the research community. Data about a protein can be retrieved by PDB ID, keyword(s), or protein sequence search. Protein domains of interest are placed close to their close homologs, facilitating analysis of closely related protein structures. Information about more distant homologs is available by browsing representatives of this protein's homology group. ECOD database emphasizes distant evolutionary relationships that otherwise cannot be found. Finally, it is the only classification of protein domain structures that is kept current with the PDB, and every structure is classified with a week delay from its release by the PDB. This feature is significant because other classifications lag behind in updates and researchers are frequently interested in the newest protein structures. Future developments of ECOD will include incorporation of protein sequences without experimentally determined structures to cover as much of the protein world as possible.
